# LSD1/KDM1A, a Gate-Keeper of Cancer Stemness and a Promising Therapeutic Target

**DOI:** 10.3390/cancers11121821

**Published:** 2019-11-20

**Authors:** Panagiotis Karakaidos, John Verigos, Angeliki Magklara

**Affiliations:** 1 Institute of Molecular Biology and Biotechnology-Foundation for Research and Technology, 45110 Ioannina, Greece; ioan_ver@yahoo.gr; 2 Biomedical Research Foundation Academy of Athens, 11527 Athens, Greece; 3 Department of Clinical Chemistry, Faculty of Medicine, University of Ioannina, 45110 Ioannina, Greece

**Keywords:** LSD1, KDM1A, lysine specific demethylase, tissue stem cells, cancer stem cells, LSD1 inhibitors, epigenetic drugs, epigenetic therapy

## Abstract

A new exciting area in cancer research is the study of cancer stem cells (CSCs) and the translational implications for putative epigenetic therapies targeted against them. Accumulating evidence of the effects of epigenetic modulating agents has revealed their dramatic consequences on cellular reprogramming and, particularly, reversing cancer stemness characteristics, such as self-renewal and chemoresistance. Lysine specific demethylase 1 (LSD1/KDM1A) plays a well-established role in the normal hematopoietic and neuronal stem cells. Overexpression of LSD1 has been documented in a variety of cancers, where the enzyme is, usually, associated with the more aggressive types of the disease. Interestingly, recent studies have implicated LSD1 in the regulation of the pool of CSCs in different leukemias and solid tumors. However, the precise mechanisms that LSD1 uses to mediate its effects on cancer stemness are largely unknown. Herein, we review the literature on LSD1’s role in normal and cancer stem cells, highlighting the analogies of its mode of action in the two biological settings. Given its potential as a pharmacological target, we, also, discuss current advances in the design of novel therapeutic regimes in cancer that incorporate LSD1 inhibitors, as well as their future perspectives.

## 1. Introduction

Epigenetic mechanisms, such as DNA methylation and post-translational histone modifications, regulate gene expression and hold a key role in reprogramming during normal development, as well as in sustaining tissue-specific transcriptional profiles. The epigenetic enzymes responsible for post-translational histone modifications can be classified into three categories: The “writers” that add chemical groups to histone tails, the “erasers” that can remove them, and the “readers” that recognize specific histone modifications and mediate their functional outcome [1]. The “erasers” encompass the histone deacetylases and lysine demethylases, which mediate modifications of the local chromatin structure and, in this manner, participate in the epigenetic regulation of gene expression [1,2]. Recent studies indicate that non-histone substrates, such as DNA, RNA, or proteins, can also be targeted by the lysine demethylases, adding another level of complexity to their role in the regulation of vital cellular processes [2]. To date, about 20 human lysine demethylases have been identified, which are divided into two subfamilies, the flavin-dependent lysine-specific demethylases (LSDs) and the 2-oxoglutarate-dependent Jumonji C-domain (JmjC) containing enzymes [2,3]. Members of the former group can demethylate only mono- or di-methylated lysine residues (see next Section 2), while the JmjC family members can also use tri-methylated lysine as a substrate [2]. The discovery of these enzymes has provided additional clues into the “puzzle” of the epigenetic regulation of several key biological processes, such as regulation of transcription, cell cycle, and cell fate determination [2,4].

Numerous studies have revealed the important role of deregulation of epigenetic mechanisms in cancer [5]. Epigenetic alterations have been identified in all tumor types and, since they are potentially reversible, much effort has been directed towards the discovery of novel therapies that could restore the normal epigenetic landscape and related gene expression patterns [6]. During recent years, histone methylation has emerged as a pivotal player in gene regulation in cancer [7]. Several studies have highlighted the role of the histone lysine demethylases in the epigenetic regulation of cancer development and progression [2] and have portrayed them as promising therapeutic targets [3].

The lysine specific demethylase 1 (LSD1) was the first histone demethylase discovered [8] and it has a dual function acting both as a co-activator and a co-repressor (see below Section 2). Having been identified initially as a regulator of embryonic stem cells (ESCs) [9], its intense study in recent years has led to the delineation of its involvement in a range of physiological processes, including cell-cycle [10], metabolism [11], and epithelial-to-mesenchymal transition [12]. At the same time, LSD1 has attracted the interest of the cancer research community, since it is upregulated in numerous malignancies and especially in aggressive and poorly differentiated tumors [13,14,15,16]. Given the central role of LSD1 in stemness, it is plausible that aberrant gene expression resulting from LSD1 dysregulation in cancer cells may be affecting pathways associated with a stem-cell phenotype. Indeed, a series of recent studies in various types of cancer have supported a primary regulatory role for the demethylase in cancer stem cells (CSCs), a small aggressive tumor subpopulation with unique properties. LSD1 has also emerged as an attractive therapeutic target; being an enzyme overexpressed in cancer, it is conceivable that its inactivation may contribute to a less malignant phenotype. 

Stem cells are present in every tissue irrespective of its developmental stage. Embryonic stem cells exist only at the early stages of development in the embryo and have the ability to differentiate towards all lineages [17]. Tissue-specific or adult stem cells appear during fetal development to support organogenesis and they remain in the body during adulthood with the ability to regenerate the tissue’s cell types [18]. Somatic cells can be reprogrammed epigenetically into induced pluripotent stem cells (iPSCs) that are considered equivalent to ESCs [19]. Rare tumor subpopulations with stem cell characteristics, such as self-renewal and capacity to differentiate, have been identified in most human neoplasias, and are known as CSCs [20]. 

The demethylase LSD1 has been studied in all the above types of stem cells. The crucial role of LSD1 in mouse and human ESCs or iPSCs has been reviewed extensively elsewhere [21,22]. In this review we aim to provide a concise picture of the LSD1-regulated networks in normal tissue stem cells and their cancerous counterparts. To this end, we have surveyed the literature solely for stem and cancer stem cell research reports that involve LSD1. We also report on ongoing clinical trials testing LSD1 pharmacological inhibitors against hematological and solid cancers and discuss the potential of its targeting in CSCs as a new therapeutic intervention.

## 2. LSD1, a Brief Overview

**Figure 1:**
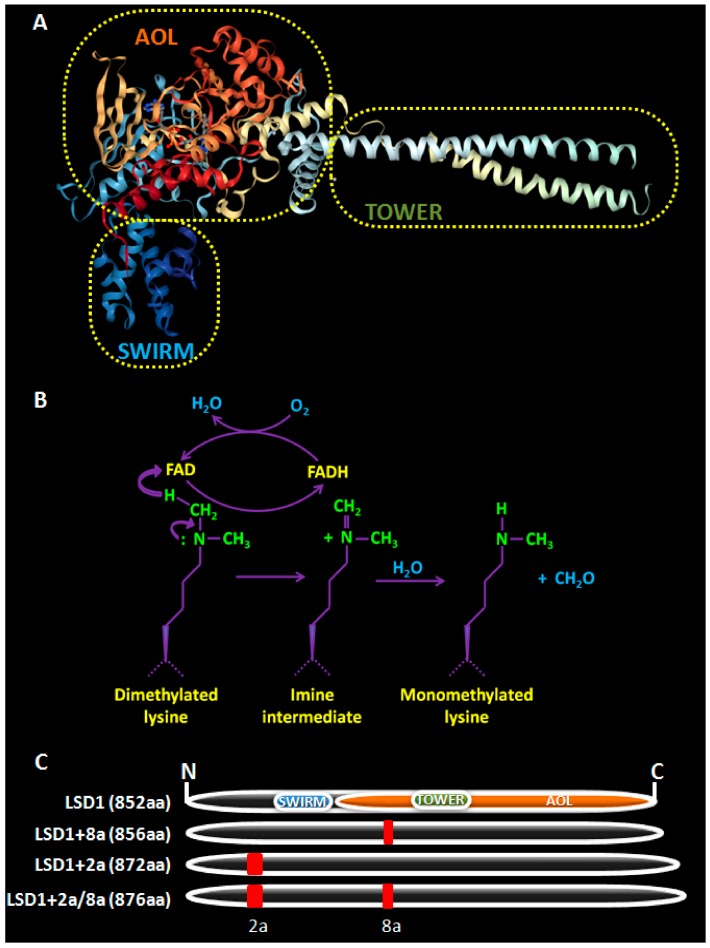
The human lysine specific demethylase 1 (LSD1) enzyme. (**A**) The crystal structure of LSD1 is depicted (image from Protein Data Bank, PDB-ID:2H94 [32,33]). (**B**) The demethylation reaction catalyzed by LSD1. The conversion of demethylated lysine to its monomethylated form is shown. (**C**) The four isoforms of human LSD1 are shown. The position of the three major domains, Swi3p/Rsc8p/Moira (SWIRM), Tower, and amine-oxidase like AOL (demethylase activity), is illustrated in the smaller, ubiquitous isoform of LSD1. The positions of exons 2a and 8a in the respective isoforms are shown in red.

The protein structure of LSD1 is highly conserved and is comprised of three major domains (Figure 1A) [2,23]. The N-terminal Swi3p/Rsc8p/Moira (SWIRM) α-helical domain of LSD1 is different than other SWIRM domains in that it cannot bind DNA, but it participates in protein-protein interactions important for its function [24]. The C-terminal amine oxidase (AO)-like domain is involved in its enzymatic activity and consists of two lobes, that form a cavity, where demethylation takes place. The first lobe contains the FAD-binding motif, which is highly conserved among all monoamine oxidases. The FAD-dependent oxidation used by the enzyme for the removal of methyl groups depends on the lone electron pair of the lysine ε-nitrogen atom restricting LSD1 from demethylating trimethylated lysine residues (Figure 1B). The second lobe forms the substrate binding sub-domain, which is larger in LSD1 than in other amine oxidases, allowing for the recognition and accommodation of the target methylated lysine as well as its neighboring residues [23]. In space, this second lobe is placed adjacently to the SWIRM domain forming a hydrophobic binding pocket that allows for further interactions with the substrates [23] and offers a structural base for the design of LSD1 inhibitors [25,26] The central Tower domain, which is not found in any other monoamine oxidases, is composed of two anti-parallel helices and it protrudes from the AO domain, serving as a docking site for LSD1’s interacting protein partners.

LSD1 exerts a context-dependent co-regulatory function in transcription (activation or repression) contingent on its association with distinct partners or multiprotein complexes (reviewed in [26]). When it is recruited by certain transcription factors, such as TLX, SNAILl, CtBP, and BRAF35 or repressive complexes (NuRD, CoREST, RCOR2, or HOTAIR/PRC2), it demethylates the “active” H3K4me1/2 marks, leading to repression of gene expression [8,21]. When it is assembled in complexes with the androgen receptor (AR) [27] or estrogen receptor (ERa) [28], respectively, LSD1 can function as a transcriptional co-activator, demethylating the “inactive” H3K9me1/2 marks, and leading to expression of hormonally-regulated genes. 

As a result of the combinatorial retention of exons 2a and 8a, LSD1 has four isoforms, with the +8a containing ones being neuro-specific, meaning that they are expressed selectively in neuronal lineages (Figure 1) [29,30]. Interestingly, it was recently shown that the LSD1+8a isoform demethylates H4K20me1, promoting in this way the transcription of neuronal-specific genes, necessary for cognitive functions, like memory and spatial learning [31].

A recently recognized important function of LSD1 is the demethylation of non-histone proteins [21]. The first such substrate identified was the tumor suppressor p53, which, upon its demethylation by LSD1 at lysine 370, losses the ability to bind to 53BP1, resulting in abrogation of the pro-apoptotic p53-mediated transcriptional activation [34]. Other non-histone substrates include E2F-1, DNMT1, MYPT1, HIF-1α, and STAT3, linking LSD1 to several major biological processes, such as DNA damage response, apoptosis, global DNA methylation, and angiogenesis [10,35,36,37,38]. 

## 3. LSD1 as a Regulator of Tissue and Cancer Stem Cells

The solid research results of a plethora of studies have established LSD1 as a critical regulator of normal hematopoiesis and neurogenesis, highlighting its involvement in the maintenance and proper differentiation of tissue stem cells. Consequently, in our review we discuss, in more detail, the findings on LSD1 functions in the stem cells of the healthy and cancerous hematopoietic and neuronal tissues, giving emphasis on the mechanisms, when these are known, and on the potential for therapeutic targeting of this demethylase. 

### 3.1. LSD1 in Tissue Stem Cells

#### 3.1.1. LSD1 in Normal Hematopoietic Stem Cells

An early study had reported the in vitro interaction of LSD1 with the GFI1/GFI1b transcription repressors that have a primary function in hematopoiesis [39]. More recently, Thambyrajah et al. elucidated the gene networks that were regulated by the transcriptional repressors GFI1 and GFI1b during embryonal hematopoiesis [40]. In mouse embryos, the first embryonic hematopoietic stem cells (HSCs) arise from the hemangioblasts, through an intermediate stage of rare endothelial cells that constitute the hemogenic endothelium, in a process called endothelial-to-hematopoietic transition [41]. At this developmental stage, Thambyrajah et al. found that GFI1/GFI1b transcription factors suppressed a large set of genes, enabling the completion of this transition that led to generation of the first HSCs and mature blood production [40]. A critical requirement for their action was the recruitment of both LSD1 and the repressive CoREST complex onto their target genes. Pharmacological inhibition of LSD1 phenocopied the effects of GFI1/GFI1b deficiency that impaired normal hematopoiesis, underscoring the significance of their interaction in this biological process [40].

This was also confirmed in adult, bone marrow-derived hematopoietic stem cells in vivo by two studies using distinct mouse models [42,43]. Sprussel et al. generated a shLSD1-based transgenic mouse model to investigate the in vivo effects of LSD1 knockdown in normal HSCs [42]. LSD1 depletion was followed by severe impairment of hematopoiesis with extensive increase in granulomonocytic, erythroid, and megakaryocytic progenitors that could not reach terminal differentiation. This was due to de-repression of GfI1b in HSCs and progenitor cells (HSPCs, lineage-committed precursors towards erythroid, megakaryocytic, and other lineages), and it was suggested that upregulation of the transcriptional repressor accounted for the failure of terminal differentiation of the above lineages [42]. In line with these results, there was a second report, where *Lsd1* was conditionally inactivated in mice [43]. This study also showed that *LSD1* was required for HSPC self-renewal. Furthermore, LSD1 was indispensable for proper differentiation both at the early and late stages of the process. Deletion of *LSD1* was associated with increased levels of H3K4me1/2 at the enhancers and transcription start sites of several stem- and progenitor-associated genes, leading to their de-repression. Interestingly, these gene expression networks involved specific Hox genes that control embryonic development. The gene expression changes were followed by severe defects in hematopoietic differentiation and terminal maturation, including the depletion of lineage-negative c-kit positive myeloid progenitor cells [43]. The authors suggested that this erroneous activation of a stem/progenitor cell signature interfered with proper differentiation, leading to impaired blood maturation. Of note, the work of Sprussel et al. [42] did not detect such a progenitor pool exhaustion, when they analyzed the long-term repopulating HSCs, however, this discrepancy might be due to the different silencing depth of the models used, i.e., *LSD1*-knockout [43] versus LSD1-knockdown [42].

Taken together, the above studies suggest that the activity of LSD1 is required in HSCs and HSPCs in order to finetune the balance between stem cell maintenance and differentiation (hematopoiesis). Dysregulation of these processes involving LSD1 is frequently observed in leukemias, as described later (3.2a). 

#### 3.1.2. LSD1 in Neural Stem/Progenitor Cells (NSPCs)

In both humans and mice, LSD1 is involved in neurogenesis through regulation of the Notch signaling pathway, which plays an essential role in the maintenance of neuronal stem cells (NSCs), and its inactivation induces their differentiation [44]. In cultures of human fetal neuronal stem progenitor cells (NSPCs), LSD1 activation epigenetically downregulated *HEYL*, a Notch target gene, and promoted neuronal differentiation [45]. In contrast, LSD1 inhibition led to suppression of neuronal differentiation and maintenance of an undifferentiated state through upregulation of *HEYL* gene expression [45]. Since *Lsd1* null embryos died at d7.5 of embryonic development, the role of the demethylase in neurogenesis could not be deciphered in those animals [36,46]. However, Lopez et al. successfully knocked down LSD1 and CoREST, through in utero electroporation, and revealed that their role was to suppress Notch signaling and to promote differentiation in the developing cerebral cortex [47]. Upon their loss, the levels of HES1, a direct Notch target, increased and those of, NGN2, a factor promoting neurogenesis, decreased, causing defects in neuronal differentiation and migration. These effects were rescued with concomitant inactivation of the Notch pathway [47]. Similar defects were also observed in pyramidal cortical neurons upon depletion of LSD1/CoREST [48]. 

Another pathway in the developing brain that is affected by LSD1 is the sonic hedgehog (Shh) signaling pathway that has diverse functions depending on the region and developmental stage, and also regulates proliferation and differentiation of NSPCs [49]. In d13.5 mouse embryos, LSD1 interacted with RCOR2 to form a co-repressor complex and epigenetically repress several genes of the Shh pathway, such as *Shh* and *Dxl2*, a step necessary for normal central nervous system development [49]. At the same developmental stage, Han et al. showed that LSD1 knockdown promoted cerebral cortical development and, in wild type embryos, the E3 ubiquitin ligase Jade-2 facilitated LSD1 targeting for degradation [50]. The regulation of LSD1 levels in the developing mouse brain is also mediated via a feedback loop that involves mir-137, which is highly expressed in the brain and controls embryonic NSPC proliferation and differentiation [51], and TLX [52], an orphan nuclear receptor with essential role in NSPC self-renewal [53]. Mir-137 negatively regulated *Lsd1* mRNA expression in embryonic NSPC, while itself was repressed by a complex of TLX with LSD1 [52]. Furthermore, LSD1 enhanced self-renewal and proliferation of ex vivo cultured NSPCs and of the mouse hippocampal dentate gyri brain region in vivo [54]. LSD1 was recruited, along with HDAC5, on the promoters of TLX downstream targets (such as *p21* and *PTEN*) and repressed their expression [54]. Collectively, the above studies suggested that LSD1 was required for the self-renewal of NSPCs and its ablation led to premature differentiation and migration during embryonic mouse cortical development [47,48,50,52]. 

In conditionally *Lsd1* knockout rat embryos, Zhang et al. observed similar migration impairment and NSPC depletion [55], as others did in mice [47,49]. In that study, LSD1 was also shown to epigenetically repress atrophin 1 (ANT1), a protein related to the autosomal dominant neuronal degenerative disease dentatorubral-pallidoluysian atrophy, and, upon its loss, NSPCs could differentiate, an effect that was rescued upon ANT1 overexpression. 

Moreover, LSD1 is involved in neurosensory differentiation. In mouse inner ear progenitors, LSD1 interacted with PAX2 and recruited the NuRD complex to regulate PAX2 target genes, enabling the maintenance of multipotent optic progenitors prior to cell fate specification [56]. In the retina, LSD1 promoted terminal differentiation of the progenitor mouse rod photoreceptors through the epigenetic control of several genes, especially those regulated by HES1 and STAT3 and their downstream signaling [57]. LSD1 was shown to play an important role in the olfactory epithelium as well, as it was strongly expressed in the multipotent globose basal cells and during tissue regeneration after methyl bromide lesions [58]. 

As discussed earlier, LSD1 has two neuro-specific isoforms (neuroLSD1), distinguished by the inclusion of the mini-exon 8a (Figure 1). In their discovery and characterization paper, Zibetti et al. also highlighted their important role in NSPCs [29]. In more detail, the LSD1+8a isoforms were expressed in all neuronal cells in rats and their levels fluctuated according to the developmental stage, with their predominance, over the other two isoforms, starting at the perinatal stage (d18.5) and lasting till post-natal day seven. Subsequently, they dropped to ~40% of total LSD1. During that period, neuroLSD1 seemed to control the timing of neurite morphogenesis, as its knockdown delayed neurite differentiation and its overexpression enhanced the premature formation of differentiated neurons. A few years later, the same group showed that phosphorylation of neuroLSD1 at threonine 369 induced a conformational change that dissociated neuroLSD1 from its co-repressor partners (CoREST, HDAC1/2), relieving morphogenetic genes from their epigenetic repression, and, thus, inducing morphogenesis in cortical neurons [59]. Further in vitro mechanistic studies in cell lines and cultures of NSPCs shed more light into its mode of action. Neuro-LSD1 was regulated by far upstream-binding protein 1 (FUBP1), a single-strand DNA- and RNA-binding protein [60], and was capable to demethylate only H3K9me2 at target genes, and this occurred in collaboration with supervillin (SVIL) [61]. 

In summary, the above studies provide strong evidence that LSD1 holds a major part in fetal neuronal development. It regulates key developmental pathways, like Notch or Shh, and contributes significantly in stem cell maintenance, as well as in the timely differentiation and migration of neuronal precursors. Moreover, its spatiotemporal activity, during major morphogenetic events, is enhanced through the expression of the neuroLSD1 isoforms in certain developmental stages.

#### 3.1.3. LSD1 in Other Normal Stem Cell Types

Even though it is best studied in the hematopoietic and neuronal systems, LSD1 has emerged as a broader regulator of tissue stemness and differentiation, as recent reports indicate. In brief, LSD1 was implicated in spermatogenesis [62,63] and adipogenesis [64,65], while its loss promoted differentiation of mesenchymal stem cells towards bone or liver [66,67,68,69,70,71]. In a different context, LSD1 was required by the satellite cells (stem cells of muscle) for myogenic regeneration after injury [72] or for proper myoblast differentiation [73]. 

All of the studies described above emphasize the prominent role of LSD1 in the maintenance of stemness and in the process of differentiation of normal stem cells. LSD1 loss attenuates the stem-cell pool both in the hematopoietic and neuronal lineage, but it abrogates terminal differentiation of the former one, while it promotes differentiation of the latter. Remarkably, the opposing regulatory functions of the enzyme exerted upon different sets of genes coordinate the desired physiological outcome at each developmental stage in a tissue-specific manner. We can speculate that this is achieved through the variable interacting partners of LSD1 that guide the enzyme to different target genes regulating cell fate.

### 3.2. LSD1 and CSCs

According to the cancer stem cell model, tumors are organized in a hierarchy, where the CSCs lie at the apex, capable of maintaining a stem-cell pool and generating more differentiated cells that form the bulk of the tumor [74]. This process draws analogies to that of normal tissue development, and there is a growing consensus that it is also driven by epigenetic mechanisms [75]. Notably, CSCs are attributed with increased resistance to conventional treatments and thus, they are considered to promote cancer recurrence and metastasis [76]. Several studies now implicate the unique chromatin state of this subpopulation in their particularly malignant properties [77,78,79]. 

**Figure 2:**
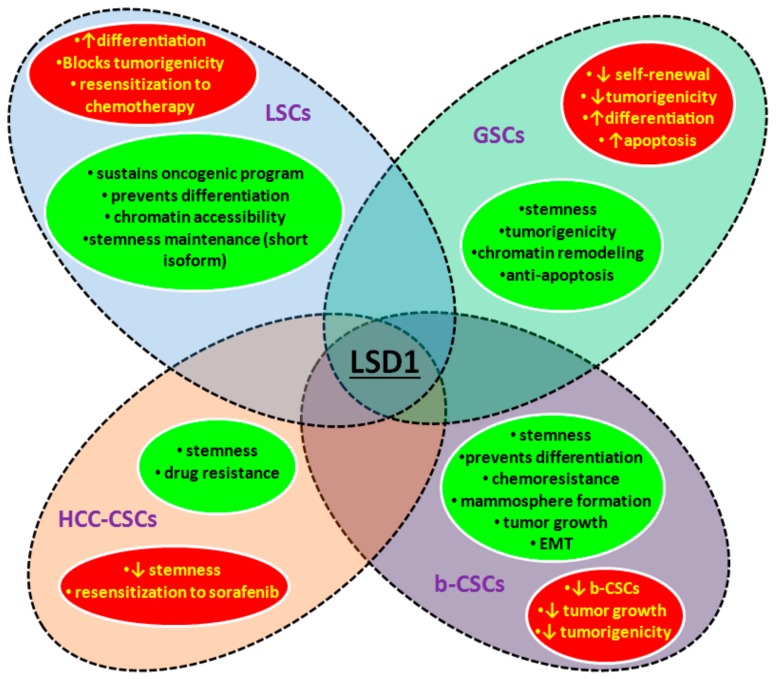
LSD1 regulates CSC properties. Key processes of CSCs are maintained and/or promoted by the function of LSD1 (shown in green circles) in the depicted types of cancer. Depletion of the enzyme affects stemness-related characteristics (shown in red circles). LSCs: Leukemic stem cells, GSCs: Glioblastoma stem cells, HCC-CSCs: Hepatocellular carcinoma CSCs, and b-CSCs: Breast CSCs (downward arrow: Decrease, upward arrow: Increase).

In the studies described next, we aim to underline the role of LSD1-dysregulated pathways in maintaining the tumor stem cell compartment and, in this way, fueling tumor growth. LSD1 has been extensively studied in leukemic and, recently, in glioblastoma SCs, and the emerging pattern is that the demethylase “locks” CSCs in an undifferentiated, therapy-resistant state. In other solid tumors, LSD1 seems to have a similar action, and recent reports have started clarifying the underlying mechanisms. Key findings of the studies discussed are summarized in Figure 2.

#### 3.2.1. LSD1 in Malignant Hematopoietic Stem Cells

An aberrant epigenetic landscape is a hallmark of several leukemias [80,81,82]. Acute myeloid leukemia (AML), the most common acute leukemia, is a prime example with recurrent mutations in several genes encoding epigenetic enzymes (e.g., methylcytosine hydroxylase TET2, histone H3K27 methyltransferase EZH2, DNA methyltransferase DNMT3A) or repeated chromosomal translocations involving the H3K4 methyltransferase mixed lineage leukemia 1 (MLL1) [80,81,82]. *LSD1* is not found mutated, but it is, frequently, overexpressed in myeloid, lymphoid, and myeloproliferative neoplasms [83], suggesting that it contributes in the pathogenesis of the disease. Several studies have supported the idea that this is achieved by enabling leukemic stem cells (LSCs) to maintain stemness, while preventing their differentiation, cell cycle arrest, and apoptosis (Figure 2). 

In one of the earliest reports, Harris et al. demonstrated that LSD1 was indispensable for the maintenance of LSC potential and it was essential in sustaining an oncogenic gene expression program necessary for tumor progression in mouse models carrying the MLL-AF9 oncogenic fusion [84]. Downregulation of this oncogenic program in LSD1-knockdown LSCs was followed by initiation of myeloid differentiation. Interestingly, LSD1 pharmacological inhibition phenocopied the knockdown effects in human patient AML cells and murine models, and it also prevented the establishment of the disease, upon murine MLL-AF9 AML cell transplantation into sub-lethally irradiated CD45.1^+^ congenic mice [84]. In line with these results, a recent work by Cusan et al. showed that LSD1 inhibition induced global accessibility onto dynamic sites of chromatin and occupancy by the myeloid transcription factors PU.1 and C/EBPα in murine MLL-F9 cells, leading to premature developmental arrest at the stage of granulocyte-macrophage progenitor [85]. Both of these studies [84,85] underlined the fact that LSD1 attenuation released LSCs from this “premature” differentiation arrest and sensitized them to anti-cancer treatments with promising results. Likewise, Schenk et al. showed that LSD1 inhibition resensitized AML cell lines or primary samples of non-acute promyelocytic leukemia AML to ATRA treatment by re-activating the all-trans-retinoic acid differentiation pathway, a therapeutic regimen that is usually ineffective in these types of leukemias [86]. 

It is noteworthy that the positive outcome of the pharmacological inhibition of LSD1 in leukemias may not be due to the inactivation of its enzymatic activity. Recent evidence suggests that certain inhibitors block the interaction between LSD1 and GFI1 and that the AML cell survival is independent of the LSD1 demethylase activity [87,88]. Maiques-Diaz et al. and Vinyard et al. supported this claim by using distinct experimental approaches [87,88]. The former observed that both LSD1 and the LSD1-K661A mutant (demethylase dead mutant) could interact with GFI1 and repress a set of enhancers, co-occupied by GFI1, LSD1, and RCOR1. These enhancers controlled key regulatory genes of monocyte/macrophage differentiation, such as *IRF8*, *KLF4,* and *MEF2C*, which were bound by the PU.1 and CEBPA transcription factors. In addition, LSD1 inactivation by the OG86 inhibitor disrupted this interaction in MLL translocation bearing AMLs, leading to activation of regulated genes within hours [87]. Vinyard et al. developed the CRISPR-superior scanning technique and mapped the effective structure-activity relationships (SARs). In other words, they mapped the mutations in the LSD1 gene that gave survival advantage to AML cells upon LSD1 inhibitor treatment, in order to gain insights into the connective links between mutation, protein function, and small molecule structure, in an effort to elucidate the mechanisms that LSD1 inhibitors use in AML [88]. They, too, concluded that LSD1’s enzymatic action was not advantageous to the AML cells, but, it was rather the interaction of LSD1 with the transcriptional repressors GFI1 and GFI1b that promoted the disease [87,88]. These findings were in agreement with Cusan et al. ([85], see above) and were also supported by the results of Maes et al., who found that, upon pharmacological LSD1 inhibition, in human AML cell lines, activation of certain genes associated with macrophage/monocytic differentiation, like *S100A12*, *ANXA2,* and *LY96*, preceded detectable changes in local histone marks or global H3K4me2 levels, leading to blast differentiation and reduced self-renewal and growth [89]. Finally, another report in acute erythroid and acute megakaryoblastic leukemia pointed towards this direction, which showed that the LSD1 inhibitor T-3775440 acted by disrupting the LSD1-Gfi1b interaction in vitro, leading to growth inhibition and differentiation; however, the authors did not address the role of LSD1’s demethylase activity in the process [90]. 

As discussed earlier, there are 4 LSD1 isoforms (Figure 1) with two of them being neuro-specific [29]. Wada et al. tried to determine the contribution of each isoform into the regulation of HSCs and LSCs and gauge the potential of LSD1 overexpression to drive malignant transformation [91]. Their results demonstrated that the shorter LSD1 isoform contributed to the enhanced stemness and self-renewal of HSCs and progenitor cells, while the neuro-specific isoforms were absent from both normal and transformed hematopoietic cells. Moreover, they found that the levels of LSD1 in normal HSCs were markedly lower than in LSCs. Finally, overexpression of the shorter LSD1 isoform upregulated several genes (including genes of the *Hox* family and *Gfi1b*) and suggested that it acted in vivo as a “founder abnormality” by driving HSCs out of quiescence and to their expansion and by generating preleukemic HSCs that were primed towards the T-cell lineage. These preleukemic HSCs were prone to develop T-cell lymphoblastic leukemia/lymphoma in a transgenic mouse model upon a second hit, such as γ-irradiation, although they admitted that, upon a different second hit, an alternative outcome may arise [91]. 

As is the case with their normal equivalents, LSCs require the activity of LSD1 for stemness maintenance but, here it also sustains their tumorigenic potential and/or drug resistance. Thus, the pharmacological inhibition of LSD1 either targeting its enzymatic activity or its interaction with other important factors provides a novel, highly promising, therapeutic approach, as also discussed later.

#### 3.2.2. LDS1 in Glioblastoma Stem Cells

Glioblastoma, the most common brain cancer in humans, is one of the deadliest human cancers and is considered incurable, despite all efforts and aggressive treatment schemes [92]. Glioblastoma cells harbor numerous genetic aberrations, but the main driving force behind tumor progression and lethality is suggested to be the CSC-subpopulation [93]. The role of LSD1 in these cells has only recently been investigated and has turned out to be a critical one for their maintenance (Figure 2).

In one of the first studies in the field, Suva et al. identified a core set of neurodevelopmental transcription factors—POU3F2, SOX2, SALL2, and OLIG2—that could reprogram the bulk of differentiated glioblastoma cells to induced stem-like tumor propagating cells [94]. Notably, RCOR2, an interacting partner of LSD1, was shown to be a target of these transcription factors and it could replace OLIG2 in the reprogramming cocktail. Knock-down of LSD1 in tumor propagating cells led to reduced cell survival, almost complete abolishment of their self-renewal capacity in vitro and loss of tumorigenicity in vivo. On the other hand, it had no effect on the proliferation or viability of differentiated glioblastoma cells. Similar results were obtained when normal human astrocytes, tumor propagating cells and differentiated glioblastoma cells were treated with S2101, a synthetic LSD1 inhibitor, which selectively affected only the cell viability of tumor propagating cells, suggesting that LSD1 could be a putative therapeutic target in tumor stem-like cells in glioblastoma patients [94]. 

The results of Sareddy et al., who investigated two LSD1 inhibitors (NCL-1 and NCD-38), also, pointed towards this direction [95]. They showed that LSD1 was overexpressed in glioblastoma stem cells (GSCs isolated from primary specimens and its inhibition reduced their self-renewal potential and viability in vitro, while it induced apoptosis and differentiation. The two inhibitors also reduced tumor growth in a mouse model of glioblastoma and specifically the NCD-38 improved overall survival. To provide a mechanism for the action of the LSD1 inhibitors, the authors examined the gene expression profile of treated GSCs and discovered induction of genes of the unfolded protein response (UPR) pathway, which is responsible for the protection of cells from hazardous misfolded proteins and protein aggregates. They suggested that UPR activation might be implicated in the phenotypic effects of LSD1 inhibition in GSCs, without, however, further substantiating their claim [95]. 

Repression of *BMP2* and *CDKN1A* has been reported to be essential for stemness maintenance of GSCs [96,97]. Two studies uncovered different mechanisms that LSD1 utilized to silence these genes and suppress differentiation [98,99]. In the first study [98] they showed that increased nuclear levels of the β isoform of glycogen synthase kinase 3 (GSK3β) were responsible for the stabilization and accumulation of LSD1 by mediating its phosphorylation, a prerequisite for its binding and subsequent deubiqitination by ubiquitin-specific protease 22 (USP22). Stabilized LSD1 repressed the expression of *BMP2*, *CDKN1A*, and *GATA6*, which in turn promoted the stemness and tumorigenicity of GSCs in vitro and in vivo, respectively. The GSK3β-USP22-LSD1 axis was validated in a cohort of clinical specimens from Grade IV GBM patients, where all three proteins showed increased levels of nuclear expression, significantly higher than their levels in a set of low-grade astrocytomas [98]. In the second study [99] the authors demonstrated that LSD1 participated in a co-repressor complex along with TLX and RCOR2 that was recruited by the SWI/SNF chromatin remodeling complex through the DPF1 and DPF3 adaptors [99]. A series of in vitro and in vivo experiments convincingly showed that this multi-protein complex played a major role in maintaining the stemness and promoting the tumor-initiating capacity of GSCs, probably through the repression of *BMP2* and *CDKN1A*.

The above studies describe a prominent loss of stemness, induction of differentiation and apoptosis and/or blockade of tumorigenicity as the key outcomes of LSD1 attenuation in GSCs. These are remarkable results especially considering that glioblastoma is fueled by the pool of cancer stem cells. Targeting these cells through LSD1 inhibition emerges as a promising therapeutic tool against a largely untreatable human cancer with a strong aberrant genetic profile. 

#### 3.2.3. LSD1 in Hepatocellular Carcinoma Stem Cells

Hepatocellular carcinoma (HCC) is one of the most common and lethal malignancies worldwide. The work of Lei et al. provided the first evidence that the LGR5^+^ HCC cells possess all typical CSC properties, including increased drug resistance [100]. LGR5^+^ cells, isolated from biopsies or cell lines, expressed higher levels of LSD1, which enhanced stemness by inducing the expression of key stemness genes, like *SOX9*, *NANOG*, *LGR5*, *CD90,* and *CD24*. On the other hand, LSD1 inhibition attenuated the expression of the aforementioned genes and the self-renewal and drug resistance potential of HCC-CSCs. Further mechanistic insights were provided when the β-catenin repressors (namely Prickle 1, APC, and Sfrp5) were identified as LSD1 targets. It was, thus, proposed that the β-catenin pathway was activated by LSD1 and regulated the stemness and chemoresistance of hepatic LGR5+ CSCs [100]. In a later study, the pharmacological inhibition of LSD1 in sorafenib-resistant HCC cells was investigated both in vitro and in vivo [101]. LSD1 inhibition downregulated the Wnt pathway through derepression of its upstream negative regulators, mentioned above, leading to reduced stemness and resensitization to sorafenib and providing a potential therapeutic approach [101].

More recently, Liu et al. confirmed several of the above results, including the contribution of high LSD1 levels to stemness and tumor growth, in a differently isolated HCC-CSC population (based on Nanog levels) [102]. Moreover, they investigated the regulation of LSD1 in HCC-CSCs and provided evidence that Notch signaling induced SIRT1 expression leading to LSD1 deacetylation, activation and stabilization. Finally, they provided in vivo evidence that the tumor microenvironment—through activated cancer-associated fibroblasts—stimulated Notch3 signaling, which, in turn, enhanced LSD1 expression to facilitate tumor growth [102]. 

Taken together, the above findings verify the prominent role of LSD1 in promoting stemness and chemoresistance in HCC-CSCs and highlight the promising therapeutic response upon its pharmacological inhibition (Figure 2). Of particular note is the last report [102], which calls attention to the fact that the contribution of the tumor microenvironment in the progress of HCC strongly involves the induction of LSD1 expression.

#### 3.2.4. LSD1 in Breast Cancer Stem Cells

Breast cancer is a frequent malignancy in the female population, strongly associated with increased mortality [103]. Although LSD1 has been extensively investigated in breast cancer, little is known about its role in breast CSCs (b-CSCs) (Figure 2). 

In contrast to the interplay of USP22 with LSD1 in GBM [98], breast cancer cells utilize USP28 to stabilize LSD1 through deubiqutination [104]. Wu et al. showed that the USP28-LSD1 axis in b-CSCS led to enhanced self-renewal and mammosphere formation in vitro, as well as increased tumor growth in vivo [104]. Interestingly, LSD1 was found to epigenetically regulate the expression of differentiation-related genes (like *p21^Cif/Waf1^*, *Hnf4*, *HoxA10*, and *FoxA2*) by directly silencing their promoters, while its effects on stemness genes (like *Sox2* and *Oct4*) was, rather, indirect [104]. In a recent report, the authors demonstrated that LSD1 knock-down nearly abolished the CD44^+^/CD24^−^ CSCs in EMT-induced breast cancer cells [105]. They also offered preliminary evidence that LSD1 was implicated in drug resistance, as they found it to be over-expressed in chemoresistant breast cancer cell lines and tumor cells that survived in mouse xenografts after prolonged docetaxel treatment and displayed increased levels of stemness genes, EMT regulators and resistance markers. Interestingly, combination treatment of mouse xenografts with the drug and an LSD1 inhibitor led to reduction of tumor volume [105]. Similar data on LSD1 regulation of b-CSCs and its therapeutic potential were also obtained by our group [106]. LSD1 knock-down and pharmacological inhibition impaired the stemness properties and significantly reduced the number of CD44^+^-CD24^-^ CSCs in vitro. These results were also confirmed in mouse xenografts, where LSD1 inhibition reduced tumor growth and led to a significant decrease of the tumor CSC-subpopulation. Orthotopic xenotransplantation of shLSD1 breast cancer cells into mice failed to lead to tumor development, suggesting that LSD1 depletion severely affected the tumor-initiating capacity of CSCs. More interestingly, by in vitro manipulation of the LSD1 levels, we showed that it conferred increased chemoresistance to breast cancer cells. Using a 3D tumorsphere system, we also demonstrated that combined administration of an LSD1 inhibitor (2-PCPA or GSK-LSD1) and a conventional drug (doxorubicin or paclitaxel) had a synergistic effect and led to a significant reduction in their number, while it targeted the CSC-subpopulation [106].

The positive contribution of LSD1 in EMT induction has been well characterized in a range of malignancies by several studies [12]. In breast cancer, LSD1 is recruited by either Snail or Slug to repress E-cadherin or BRCA1 and promote EMT and metastasis, and its phosphorylation at serine 112 by PKCα is a crucial step in this process [107,108,109,110]. While investigating the role of UTX in b-CSCs, Choi et al. revealed that it co-operated with LSD1/HDAC1, in order to silence EMT-transcription factors, competing against the c-Myc/MLL4 complex [111]. In this study, the UTX expression levels, which are usually downregulated in cancers, were the critical factor for the negative regulation of EMT by LSD1. Specifically, UTX facilitated the binding of LSD1 onto EMT-transcription factor promoters, where MLL4 was consistently bound. When UTX levels were high, LSD1 was recruited and outcompeted MLL4-activating methylation leading to EMT repression, while low UTX expression prevented LSD1 binding and sustained EMT progression [111].

The above studies provide another prime example of the contribution of LSD1 in maintaining tumor subpopulations in an undifferentiated and chemoresistant state. As in previous cases, there is a persistent pattern that LSD1 inhibition can be of therapeutic value and, in combination with standard chemotherapeutic drugs, could provide a more effective alternative in the fight against breast cancer.

#### 3.2.5. LSD1 in Other Types of CSCs

A limited number of studies have been performed in other types of CSCs, they confirm, however, the link between LSD1 and cancer stemness. In colorectal carcinoma, there is a positive correlation between the immunohistochemical expression of LSD1 with LGR5, an established stem cell marker of this tissue [112]. Stem cell derived tumors, such as embryonic carcinomas, teratocarcinomas and seminomas, are exceptionally vulnerable to LSD1 inhibition with severe growth impairment [113,114]. Moreover, in ovarian teratocarcinoma cells, LSD1 inactivation promoted CSC differentiation [115]. Finally, it was reported that LSD1 regulated the expression of SOX2, which controlled growth, differentiation and survival of a range of putative CSCs of breast, lung, prostate and ovarian origin [116]. 

The studies described in this section provide compelling evidence for the contribution of LSD1 in the maintenance of the tumor CSC-compartment, however, little is, still, known about the associated gene networks it regulates. The other valid argument put forth by these studies is the therapeutic value of LSD1 blockade (discussed next Section 4). Despite the intense research in the field of discovery of LSD1 pharmacological inhibitors, only recently have we started getting a better grasp of their therapeutic mode of action. It appears that disruption of the non-enzymatic function of LSD1 may be equally important as the inhibition of its demethylase activity in producing therapeutic effects both in leukemias [87,88] and in solid tumors [117,118].

## 4. LSD1 as a Therapeutic Target in Cancer Treatment

Epigenetic dysregulation is now believed to be a hallmark of cancer [119] and numerous epigenetic inhibitors have been developed and have been undergoing clinical trials [120,121], in the hope of developing novel, more successful therapies. However, the first generation of epigenetic drugs (non-selective DNMT and HDAC inhibitors) were poorly effective in solid tumors and, also, highly toxic [121]. Since then, more active and selective ones have been produced and administration schemes have been optimized [120,121]. In more detail, novel DNMT inhibitors have become safely effective after dozing optimization in recent years [120,121]. The highly toxic, old HDAC inhibitors have been replaced by second generation ones with increased selectively, targeting distinct isoforms, since the toxicity of the initial ones was attributed to their broad activity across isoforms [120,121]. Preclinical and clinical studies suggest that combinatorial regimes, involving epigenetic drugs and conventional chemotherapy or immunotherapy, could produce an additive therapeutic outcome [83,120,121]. 

**Figure 3:**
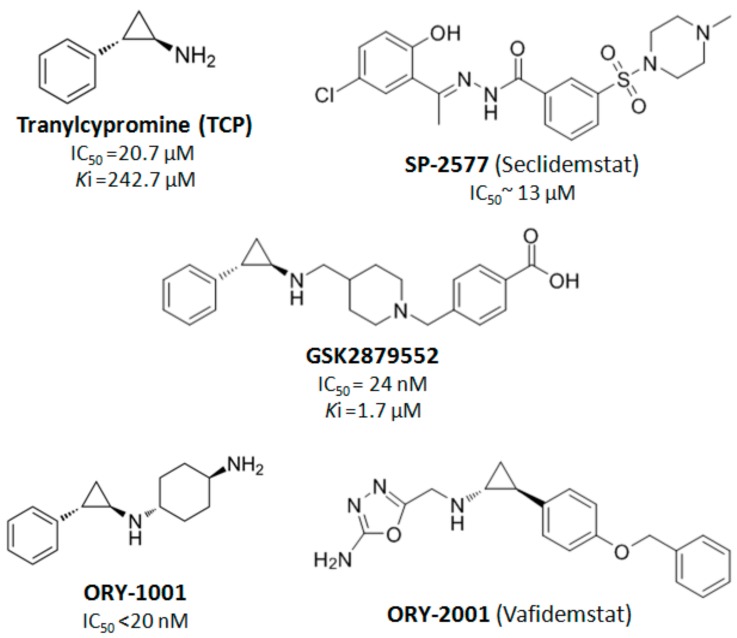
An overview of the available LSD1 inhibitors under clinical evaluation (data retrieved from [122,123] and tranylcypromine [124], ORY-1001 [89], GSK2879552 [125], and SP-2557 [126]).

Regarding LSD1 inhibitors, the FDA-approved anti-depressant Tranylcypromine was a broad monoaminoxidase (MAO) inhibitor [83,120,121,122,123]. Recent efforts have generated several derivatives of Tranylcypromine with reduced MAO-inhibitory activity and increased LSD1 specificity [25,122,123]. Chemical modifications of the (±) *trans*-2-phenylcyclopropylamine (2-PCPA or parnate or tranylcyclopromine) scaffold has been used for the development of most of the LSD1 inhibitors under clinical evaluation (e.g., the *N*-alkylated 2-PCPA derivatives ORY-1001, GSK2879552 and ORY-2001 shown in Table 1 and Table 2). In addition, several other small molecules have been synthesized as potent LSD1 inhibitors and they have exhibited safer metabolic profiles in preclinical and clinical studies, such asthe SP-2577 (Seclidemstat) synthetic inhibitor [83,120,121,122,123]. The available structures and effectiveness/potency values of these LSD1 inhibitors are given in Figure 3. These inhibitors are currently used in clinical trials in hematological (Table 1) and solid cancers (Table 2) as discussed next Section 5.

A paradigm of the therapeutic benefit that can be derived from the targeting of LSD1 emerges from the preclinical studies in leukemias discussed earlier. These findings underscore the need to block LSD1‘s function in LSCs as a means to achieve a better therapeutic response. Administration of LSD1 inhibitors as a monotherapy [84,85,87,90], or in combination with other epigenetic or anti-cancer drugs [86,89] seem to yield promising therapeutic results. Interestingly, such schemes are now being tested in the clinic against various types of leukemias (Table 1).

Similarly, in the solid tumor preclinical reports covered in this review, treatment schemes with LSD1 inhibitors alone [95,101,115] or in combination with other drugs [105,106,130] produced impressive results with therapeutic potential that is reflected in the long list of ongoing clinical trials in a range of human cancers (Table 2). 

Since LSD1 depletion seems to affect the CSC pool, it is reasonable to assume that, at least part of the therapeutic effects observed in these clinical trials, are due to targeting of these cells. However, it should be pointed out that these clinical studies do not include functional assays that can assess this parameter yet. Similarly, in most of the pre-clinical studies on LSD1 inhibition [83,131,132] the outcome is usually evaluated through its bulk characteristics i.e., tumor growth/volume, survival, dosing, and toxicity, leaving the effect on the CSC content elusive, with a few exceptions [105,106]. In our opinion, the solid findings provided by the basic research reports we presented herein prove that the regulation of CSCs by LSD1 is an important aspect of its role in cancer and it should be evaluated in all preclinical and clinical future studies. 

Irrespective to the exact molecular mechanisms, it is a fact that LSD1 inhibition suppresses disease progression and therefore, raises hopes for more effective cancer treatments. In this context, novel approaches and combinatorial schemes that involve LSD1 inhibitors are underway. For example, numerous novel inhibitors (called chimeric or hybrid) have been designed, so that single molecules can be directed against multiple targets [133]. This new class of epigenetic inhibitors includes the LSD1/HDAC hybrid inhibitors that target simultaneously LSD1 and HDAC1 or HDAC1/2 [134,135,136]. Such drugs guarantee the combined effect at multiple targets in the very same cell, apart from the more favorable pharmacokinetic or pharmacodynamic parameters or their more predictable and less complex metabolism [133]. Other novel therapeutic approaches involve combinatorial treatment regimens that utilize epigenetic agents to prime the activity of the second agent [121]. As such, LSD1 inhibition in non- acute promyelocytic leukemia AML promotes myeloid differentiation, which facilitates an ATRA-driven therapeutic response [86], which, otherwise, was ineffective (see 3.2a). Remarkably, some of them have reached clinical evaluation (Table 1 and Table 2). 

## 5. Conclusions

Since the discovery of the epigenetic regulation of gene expression, a new level of complexity has emerged in numerous research fields, spanning from stem cell and developmental biology to cancer biology. Epigenetic mechanisms can reprogram a terminally differentiated cell to iPSC, or a healthy cell to a malignant one without permanent genetic alterations (e.g., pediatric tumors like ependymona [137]). Given the largely common biological background of healthy and cancerous tissues, the lessons learned from normal physiological processes, such as development, can be applied to the discovery of the deregulated pathways in malignancies. This review tried to emphasize this fact and to highlight the similarities of LSD1 function in healthy and malignant SCs, since their maintenance seem to be subject to common biological processes, albeit deregulated in the case of cancer. The general notion that LSD1 enhances stemness in normal stem and progenitor cells is fully preserved in CSCs, where the same genes/pathways are in play, but deregulated. For example, the interplay between LSD1 and the GFI transcription factors during normal hematopoiesis is also seen in leukemias. The TLX-LSD1/RCOR2 axis is functional both in NSPCs and GSCs, where it positively regulates stemness gene expression (Sox2, Oct4). Finally, the frequently promoting effect of LSD1 inhibition on the differentiation of normal SCs is increasingly used as a therapeutic approach in the most undifferentiated and aggressive human malignancies (AML, glioblastoma, or HCC) [121,131,132] with promising preclinical outcomes, validating their entry into clinical trials (Table 1 and Table 2).

## Figures and Tables

**Table 1:** List of clinical trials with LSD1 inhibitors in human blood and bone marrow malignancies [127,128,129].

Code	Title	Status	Conditions	Interventions
DRKS00006055	Phase I/II study of sensitization of non-M3 acute myeloid leukemia (AML) blasts to all-trans retinoic acid (ATRA) by epigenetic treatment with tranylcypromine (TCP), an inhibitor of the histone lysine demethylase 1 (LSD1)	Recruiting	AML and Myelodysplastic syndrome	TCP| ATRA| AraC
EUCTR2013-002447-29	A phase I study of Human Pharmacokinetics and Safety of ORY-1001, and LSD1 inhibitor, in relapsed or refractory acute leukaemia (AL)	Not Recruiting	Refractory or Relapsed acute leukaemia	ORY-1001
EUCTR2018-000482-36	A pilot study to assess the safety, tolerability, dose finding and efficacy of ORY-1001 in combination with azacitidine in older patients with AML in first line therapy	Ongoing	AML	ORY-1001|Etoposide| Carboplatin| Cisplatin
NCT02717884	Study of Sensitization of Non-M3 AML Blasts to ATRA by Epigenetic Treatment With Tranylcypromine (TCP)	Recruiting	AML and Myelodysplastic Syndrome	TCP| ATRA| cytarabine
NCT02842827	IMG-7289, With and Without ATRA, in Patients With Advanced Myeloid Malignancies	Completed	AML and Myelodysplastic Syndrome	IMG-7289| ATRA
NCT03136185	IMG-7289 in Patients With Myelofibrosis	Recruiting	Myelofibrosis| PPV-MF| PET-MF| PMF	IMG-7289

**Table 2:** List of clinical trials with LSD1 inhibitors in human solid tumors [127,128,129].

Code	Title	Status	Conditions	Interventions
NCT03514407	A Study of INCB059872 in Relapsed or Refractory Ewing Sarcoma	Recruiting	Relapsed Ewing Sarcoma	INCB059872
NCT03600649	Clinical Trial of SP-2577 (Seclidemstat) in Patients With Relapsed or Refractory Ewing Sarcoma	Recruiting	Ewing Sarcoma	SP-2577
EUCTR2018-000469-35	A pilot study to assess the safety, tolerability, dose finding and efficacy ORY-1001 in combination with platinum-etoposide chemotherapy in patients with relapsed, extensive-stage disease small cell lung cancer	Ongoing	Relapsed, extended-stage disease small cell lung cancer	ORY-1001
EUCTR2017-002838-23	Randomized, double-blind, placebo-controlled, 3-arm, 36 weeks parallel-group study to evaluate the safety and tolerability of ORY-2001 in patients with Relapsing-Remitting Multiple Sclerosis and Secondary Progressive Multiple Sclerosis	Authorised	Relapsing-Remitting Multiple Sclerosis and Secondary Progressive Multiple Sclerosis	ORY2001
NCT03505528	An Early Phase Study of Abraxane Combined With Phenelzine Sulfate in Patients With Metastatic or Advanced Breast Cancer	Recruiting	Metastatic Breast Cancer	Nanoparticle albumin-bound paclitaxel| Phenelzine Sulfate
NCT02712905	An Open-Label, Dose-Escalation/Dose-Expansion Safety Study of INCB059872 in Subjects With Advanced Malignancies	Recruiting	Solid Tumors and Hematologic Malignancy	INCB059872| ATRA| azacitidine| nivolumab
NCT03895684	Phase 1 Trial of the LSD1 Inhibitor SP-2577 (Seclidemstat) in Patients With Advanced Solid Tumors	Not yet recruiting	Advanced Solid Tumors	SP-2577 Seclidemstat
